# Stability of 3-bromotyrosine in serum and serum 3-bromotyrosine concentrations in dogs with gastrointestinal diseases

**DOI:** 10.1186/s12917-015-0321-0

**Published:** 2015-01-17

**Authors:** Panpicha Sattasathuchana, Niels Grützner, Rosana Lopes, Blake C Guard, Jan S Suchodolski, Jörg M Steiner

**Affiliations:** Gastrointestinal Laboratory, College of Veterinary Medicine, Texas A&M University, College Station, Texas 77843 USA; Department of Companion Animal Clinical Sciences, Faculty of Veterinary Medicine, Kasetsart University, Bangkok, 10900 Thailand; Department for Clinical Veterinary Medicine, Clinic for Swine, University of Bern, Bern, 3012 Switzerland

**Keywords:** 3-bromotyrosine, Canine, Eosinophilic gastroenteritis, Stability

## Abstract

**Background:**

3-Bromotyrosine (3-BrY) is a stable product of eosinophil peroxidase and may serve as a marker of eosinophil activation. A gas chromatography/mass spectrometry method to measure 3-BrY concentrations in serum from dogs has recently been established and analytically validated. The aims of this study were to determine the stability of 3-BrY in serum, to determine the association between peripheral eosinophil counts and the presence of an eosinophilic infiltrate in the gastrointestinal tract, and to compare serum 3-BrY concentrations in healthy dogs (n = 52) and dogs with eosinophilic gastroenteritis (EGE; n = 27), lymphocytic-plasmacytic enteritis (LPE; n = 25), exocrine pancreatic insufficiency (EPI; n = 26), or pancreatitis (n = 27).

**Results:**

Serum 3-BrY concentrations were stable for up to 8, 30, and 180 days at 4°C, −20°C, and −80°C, respectively. There was no significant association between peripheral eosinophil count and the presence of eosinophils in the GI tissues (*P* = 0.1733). Serum 3-BrY concentrations were significantly higher in dogs with EGE (median [range] = 5.04 [≤0.63-26.26] μmol/L), LPE (median [range] = 3.60 [≤0.63-15.67] μmol/L), and pancreatitis (median [range] = 1.49 [≤0.63-4.46] μmol/L) than in healthy control dogs (median [range] = ≤0.63 [≤0.63-1.79] μmol/L; *P* < 0.0001), whereas concentrations in dogs with EPI (median [range] = 0.73 [≤0.63-4.59] μmol/L) were not different compared to healthy control dogs.

**Conclusions:**

The present study revealed that 3-BrY concentrations were stable in serum when refrigerated and frozen. No relationship between peripheral eosinophil count and the presence of eosinophils infiltration in the GI tissues was found in this study. In addition, serum 3-BrY concentrations were increased in dogs with EGE, but also in dogs with LPE and pancreatitis. Further studies are needed to determine whether measurement of 3-BrY concentrations in serum may be useful to assess patients with suspected or confirmed EGE or LPE.

## Background

Chronic enteropathy (CE) is characterized by recurrent or persistent gastrointestinal (GI) signs for more than 3 weeks [1,2]. The diagnostic process for patients with suspected CE requires exclusion of GI parasites and other extra GI diseases (e.g., pancreatitis, exocrine pancreatic insufficiency (EPI)). CE is classified by a patient’s response to a given treatment trial, namely, food-responsive diarrhea, antibiotic-responsive diarrhea, and steroid-responsive diarrhea. The histological findings in dogs with steroid-responsive diarrhea (idiopathic inflammatory bowel disease; IBD) differ due to various inflammatory cells within the intestinal mucosa. IBD in dogs can thus be subclassified into eosinophilic gastroenteritis (EGE), lymphocytic-plasmacytic enteritis (LPE), granulomatous enteritis, and histiocytic ulcerative colitis [3,4]. In dogs with IBD, the small intestine is affected more commonly than the large intestine [1]. Clinical scoring systems including the canine chronic enteropathy clinical activity index (CCECAI) and the canine IBD activity index (CIBDAI) can be used to evaluate the disease severity and treatment response in dogs with IBD [1,5]. Nevertheless, no biomarker for eosinophilic infiltration has previously been identified in dogs with GI disease.

The defining feature of EGE is infiltration of eosinophils in the GI tract. EGE can be caused by parasite infestation, neoplasia, allergy, IBD, or hypereosinophilic syndrome. Although previous studies have reported that the histopathological findings of GI biopsies would suggest that EGE is secondary to LPE in canine IBD [6,7], eosinophils were shown to play crucial roles in stimulating inflammation and motility, leading to clinical signs such as diarrhea, inflammation, tissue destruction, fibrosis formation, and/or strictures [8,9]. Therefore, a marker of eosinophil activation may provide a useful tool for evaluating the contribution of eosinophils in canine CE.

Eosinophil peroxidase (EPO) is a potent granular cytotoxic heme-protein released during the activation of eosinophils [10-12]. Under physiological conditions, EPO utilizes bromide to yield hypobromous acid (HOBr) [13]. Bromination of tyrosine by HOBr in surrounding tissues and blood occurs rapidly and results in the production of 3-bromotyrosine (3-BrY) [10-12,14]. Structurally and physiologically, 3-BrY is a stable product. Therefore, it can be used as a noninvasive marker of eosinophil-catalyzed protein oxidation. A method to measure 3-BrY in dog serum using an electron ionization gas chromatography/mass spectrometry (EI-GC/MS) has recently been developed and analytically validated [15].

The hypothesis of this study was that activation of EPO plays an important role in the pathogenesis of EGE and serum concentrations of 3-BrY may have diagnostic potential as a specific biomarker for EGE. The assay may be particularly useful to differentiate between patients with EGE and LPE, as well as dogs with other GI diseases. The objectives of this study were (1) to assess the stability of serum 3-BrY concentrations after storage at 4°C, −20°C, and −80°C, (2) to determine the association between peripheral eosinophil counts and the presence of an eosinophilic infiltrate in the GI tract, and (3) to compare serum 3-BrY concentrations between healthy control dogs and dogs with various GI diseases, such as EGE, LPE, EPI, and pancreatitis.

## Methods

### Determination of stability for 3-BrY in serum samples

Excess serum samples that were submitted to the Gastrointestinal Laboratory (GI Lab) at Texas A&M University for diagnostic purposes were pooled. Known quantities of pure 3-BrY (BOC Science Company, Shirley, NY) were mixed to pool serum samples to obtain 10 different 3-BrY concentrations within the working range of the 3-BrY assay (0–50 μmol/L). On day 0, samples were prepared, divided into aliquots, and stored at 4°C, −20°C, or −80°C until analysis. Serum 3-BrY concentrations were determined on days 0, 2, 8, 16, 30, 60, and 180. On day 0, samples were analyzed immediately after they were prepared. Serum samples stored at 4°C were analyzed on days 2 and 8. Serum samples stored at −20°C were analyzed on days 8, 16, 30, and 60. Finally, serum samples stored at −80°C were analyzed on days 16, 30, 60, and 180.

### Determination of the association between peripheral eosinophil counts and the presence of an eosinophilic infiltrate in the gastrointestinal tract

Peripheral eosinophil counts were records in dogs with EGE and dogs with LPE. 25 dogs with EGE were enrolled based on the presence of eosinophilic infiltrates in GI biopsy specimens. 23 dogs with LPE were enrolled based on the presence of lymphocytes and plasma cells infiltrate in GI biopsy specimens.

### Comparison of serum 3-BrY concentrations between healthy control dogs and dogs with various gastrointestinal diseases

#### Samples from healthy control dogs

Serum samples from 52 healthy control dogs were collected. The sample collection protocol was approved by the Texas A&M University Institutional Animal Care and Use Committee (#2012-101), and informed owner consent was obtained for all dogs. None of the healthy dogs did manifest any clinical or laboratory abnormalities and received regular vaccinations and deworming.

#### Samples from dogs with EGE

Surplus canine serum samples from submissions to the GI Lab at Texas A&M University from 27 dogs with EGE were used for this study. The diagnosis was based on the presence of eosinophilic infiltrates in GI biopsy specimens. All patients had serum cPLI and cTLI concentrations within the reference interval.

#### Samples from dogs with LPE

Surplus canine serum samples from submissions to the GI Lab at Texas A&M University from 25 dogs with LPE were used. The diagnosis of LPE was based on the presence of lymphocytes and plasma cells with an absence of eosinophils in the GI mucosa during histological evaluation of GI biopsies. All patients had serum cPLI and cTLI concentrations within the reference interval.

#### Samples from dogs with EPI

Surplus canine serum samples from submissions to the GI Lab at Texas A&M University from 26 dogs with a serum canine trypsine like immunoreactivity (cTLI) concentration ≤2.5 μg/L were used. All of these samples showed a normal serum canine pancreatic lipase immunoreactivity (cPLI) concentration and an undetectable serum cobalamin concentration ≤149 ng/L.

#### Samples from dogs with pancreatitis

Surplus canine serum samples from submissions to the GI Lab at Texas A&M University from 27 dogs with a serum cPLI concentration ≥1001 μg/L were used. It should be noted that the suggested diagnostic cut-off value of serum cPLI concentration for a diagnosis of pancreatitis is >400 μg/L. Also, all patients had serum cobalamin and cTLI concentrations within the respective reference interval.

#### Samples from dogs with CE

Dogs with CE comprised of 25 dogs with LPE and 27 dogs with EGE.

All serum samples were stored at −80°C for up to 6 months until analysis. Population demographics for dogs enrolled in this study are shown in Table 1. Standard questionnaires were sent out to the primary care veterinarian to obtain histories, clinical signs at the time of sample collection, and the final diagnosis. Questionnaires for 25 (92.6%) dogs with EGE, 23 (92.0%) dogs with LPE, 14 (53.8%) dogs with EPI, and 15 (55.6%) dogs with pancreatitis were completed by the primary care veterinarian. The clinical diagnosis for each dog was based on the result of the histological evaluation of GI biopsies or clinical laboratory data such as cTLI and cPLI. The tissues that had been evaluated for the purpose of this study included stomach, duodenum, ileum, and colon. However, there was no uniformity as to the sample type evaluated in all patients. Also, left-over serum samples were used from dogs with histopathological reports to indicate the presence of either LPE or EGE.

**Table 1:** **Population demographics for dogs enrolled in this study**

	**Healthy dogs**	**EGE**	**LPE**	**EPI**	**Pancreatitis**
Number of dogs	52	27	25	26	27
Age^*^ (years)	4.0 (1–10)	5.0 (<1-12)	8.5 (2–12)	5.8 (1–16)	11.0 (<1-16)
Sex					
Male	61.5% (n = 32)	44.4% (n = 12)	52.0% (n = 13)	42.3% (n = 11)	48.1% (n = 13)
Female	39.2% (n = 20)	55.6% (n = 15)	48.0% (n = 12)	57.7%(n = 15)	52.9% (n = 14)
Breed sizes					
Small (<10 kg)	23.1% (n = 12)	18.6% (n = 5)	56.0% (n = 14)	23.1% (n = 6)	59.3% (n = 16)
Medium (10–20 kg)	42.3% (n = 22)	33.3% (n = 9)	20.0% (n = 5)	23.1% (n = 6)	7.4% (n = 2)
Large (>20 kg)	34.6% (n = 18)	48.1% (n = 13%)	24.0% (n = 6)	53.8% (n = 14)	33.3% (n = 9)

^*^Median (minimum to maximum range).

### Measurement of serum 3-BrY

#### Preparation of internal standards

D_3_-bromotyrosine (D_3_-BrY) was used as an internal standard and prepared by reacting d_4_-L-tyrosine (Cambridge Isotope Laboratories, Inc., Tewksbury, MA) with N-bromosuccinimide (Sigma-Aldrich, St. Louis, MO) in water [12,16,17]. D_3_-BrY was isolated by reverse-phase high performance liquid chromatography (HPLC) using a C18 HPLC column (Phenomenex, Torrance, CA). Purified D_3_-BrY fractions were collected and stored at −80°C under helium until use.

#### Measurement of 3-BrY in canine serum

The sample preparation protocol was adapted from previous publications [10,16,18]. Eight nanomoles (16 μmol/L) of D_3_-BrY were added in a 1 to 2 dilution mixture of water (250 μl) and serum sample (250 μl). The volume of the mixture was adjusted to 2 mL with 0.1% trifluoroacetic acid (Sigma-Aldrich, St. Louis, MO), pH5.0 and centrifuged at 4°C for 10 min at 16,000 × g. After centrifugation, the mixture was passed through a C18 solid phase extraction column (Sigma-Aldrich Company, St. Louis, MO). 3-BrY was eluted from the column with 25% methanol (Sigma-Aldrich, St. Louis, MO) in water. The eluent was immediately dried in a rotary vacuum device (Eppendorf, Hauppauge, NY) at 45°C and stored at −80°C until further analysis.

The derivatization protocol was modified from protocols described previously [10,16,18]. The previously dried sample was mixed with 100 μL of acetonitrile (Thermo Fisher Scientific, Inc., Pittsburgh, PA) and 40 μL of diisopropylethylamine (Sigma-Aldrich, St. Louis, MO). The sample was incubated on ice for 5 min. Ethyl heptafluorobutyrate (Sigma-Aldrich, St. Louis, MO) was added into the sample and the sample underwent an incubation period for 30 min on ice. Then, the sample was sonicated in a water bath for 1 h at room temperature. Excess reagents were evaporated under a nitrogen stream at room temperature. Thirty μL of N-methyl-N-(t-butyldimethylsilyl)-trifluoroacetamide (MtBSTFA) (Thermo Fisher Scientific, Inc., Pittsburgh, PA) was added to the sample followed by 30 min incubation at room temperature. The sample was completely dried under a nitrogen stream and redissolved in 50 μL of undecane (Sigma-Aldrich, St. Louis, MO) containing 25% (v/v) MtBSTFA. One μL of the clear supernatants were analyzed immediately by EI-GC/MS.

An Agilent 7890A gas chromatography (Agilent Technologies, Santa Clara, CA) and a 5975C mass detector (Agilent Technologies, Santa Clara, CA) were used to measure 3-BrY concentrations. A capillary column (Agilent Technologies, Santa Clara, CA) was used to separate the analytes using helium as a carrier gas. The injector, transfer line, and source temperature were initially set at 180°C, 300°C, and 250°C, respectively. The oven temperature gradient was increased at a rate of 40°C/min from 180°C to 310°C. Ions were monitored at m/z 257 and 259 for 3-BrY and D_3_-BrY, respectively.

### Statistical methods

All statistical analyses were performed with commercial software packages, JMPPro 10 (SAS Institute Inc., Cary, NC) or GraphPad PRISM5.0 (GraphPad software, Inc. La Jolla, CA). Each data set was tested for normality using a Shapiro-Wilk’s test. To evaluate stability, serum 3-BrY concentrations for fresh samples and samples at each of the 3 temperatures (4°C, −20°C, and −80°C) were compared using repeated measures ANOVA. The Dunn’s post-test was used to determine the differences in serum 3-BrY concentrations at different time points under the same storage temperature. In addition, the coefficient of variation (%CV = [standard deviation/mean] × 100) was used to determine the variability of serum 3-BrY concentration under each storage condition.

A Fisher’s exact test was used to determine the association between peripheral eosinophil count and the presence of eosinophils in the GI tract in dogs with EGE and dogs with LPE. A Kruskal-Wallis test was used to evaluate the difference of serum 3-BrY concentrations between healthy dogs and dogs with various GI diseases. The Dunn’s post-test was applied to determine differences between groups. The Mann–Whitney *U* test was used to determine the differences between dogs with CE and healthy control dogs. For all analytical tests, significance was set at *P* < 0.05.

## Results

**Figure 1:**
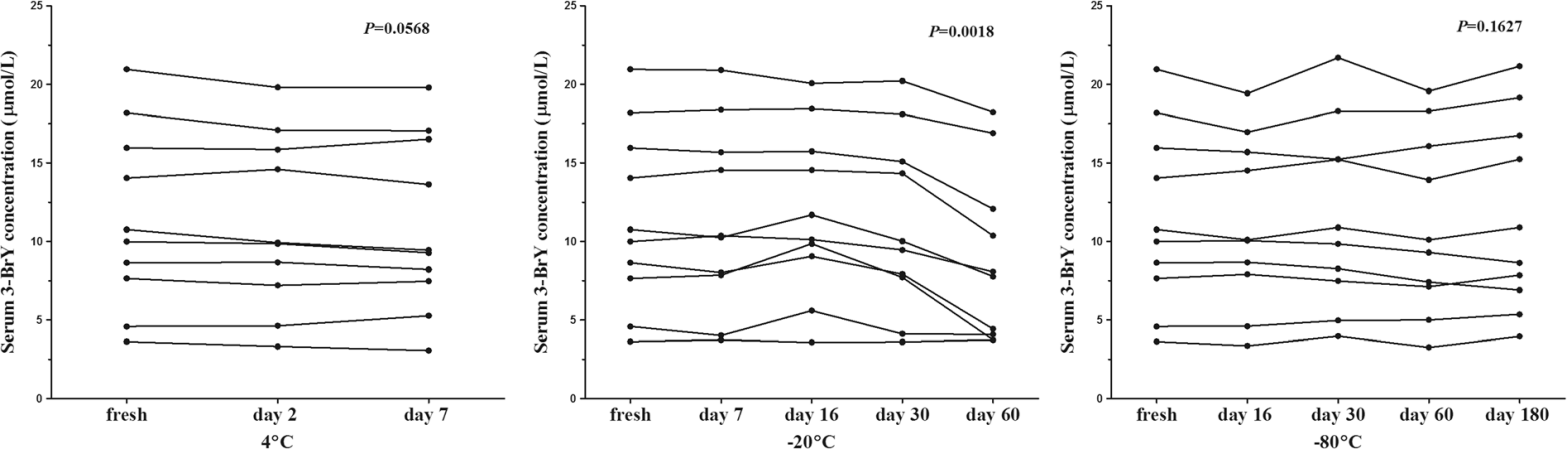
**Stability of serum 3-BrY concentration for each storage temperature.** When compared to fresh samples, 3-BrY concentrations were stable at 4°C for up to 7 days (%CV ≤ 8.5, *P* > 0.05), at −20 up to 30 days (%CV ≤ 15.8, *P* > 0.05 ), and at −80°C up to 180 days (%CV ≤ 9.9, *P* > 0.05). However, 3-BrY concentrations were not stable at −20°C at 60 days (%CV ≤ 30.3, *P* = 0.0018).

No significant differences were found between the mean serum 3-BrY concentrations for 10 individual samples stored at 4°C, −20°C, or −80°C for ≤8 days, ≤30 days, or ≤180 days, respectively (Figure 1). However, the Dunn’s post test showed that 3-BrY was not stable after 60 days at −20°C (*P* = 0.0018). The mean and %CV for each sample in each storage condition were displayed in Table 2.

**Table 2:** **Mean and %CV of 3-BrY concentrations for each pooled canine serum sample (n = 10) when measured fresh or stored under different storage conditions (4°C for 7 days, −20°C for 60 days, and −80°C for 180 days)**

**Sample**	**Mean 3-BrY ± SD (μmol/L)**	**%CV**		
		**Fresh and 4°C**	**Fresh and −20°C**	**Fresh and −80°C**
1	3.9 ± 1.3	8.5	14.4	9.1
2	4.7 ± 0.6	7.9	2.0	6.6
3	7.4 ± 1.4	3.0	14.8	4.2
4	7.6 ± 1.4	2.9	24.2	9.9
5	9.7 ± 0.9	4.0	30.3	6.3
6	10.3 ± 1.0	6.6	9.6	3.9
7	13.9 ± 1.5	3.5	13.3	4.3
8	15.5 ± 1.2	2.2	10.9	3.5
9	17.8 ± 0.8	3.6	3.5	4.3
10	19.8 ± 1.6	3.3	5.5	4.9

**Figure 2:**
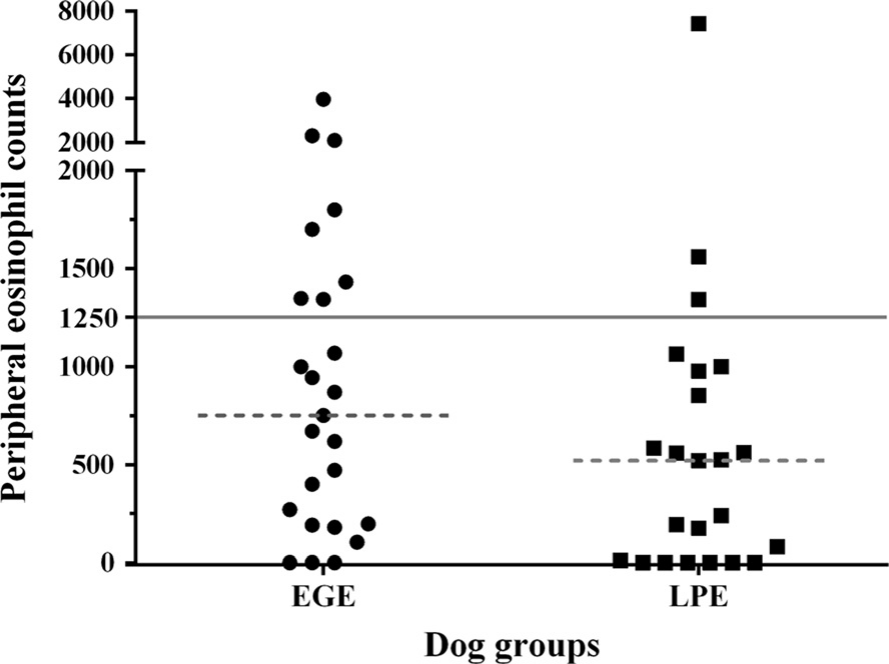
**Scatter plot of peripheral eosinophil counts in dogs with EGE compared to dogs with LPE.** There was no association between peripheral eosinophil counts and eosinophils infiltration in the GI tissues (*P* = 0.1733). The medians of peripheral eosinophil counts for dogs with EGE and LPE were 751 and 520 cell/μl, respectively (dashed lines). The horizontal solid line represents the upper limit of peripheral eosinophil.

**Figure 3:**
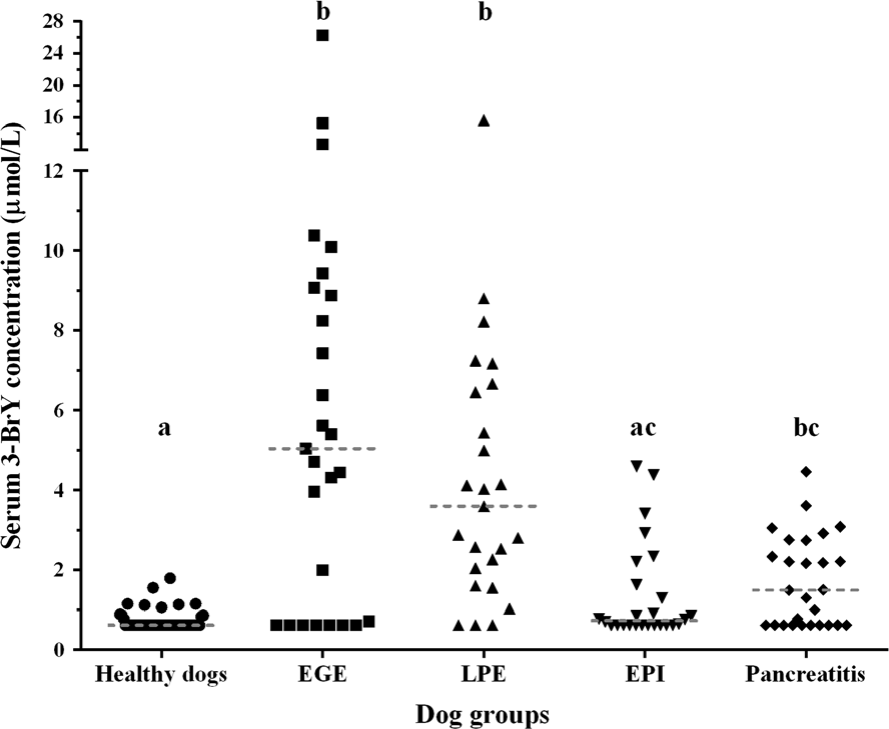
**Serum 3-BrY concentrations in healthy dogs (n = 41), dogs with EGE (n = 27), LPE (n = 25), EPI (n = 26), or pancreatitis (n = 27).** The medians of serum 3-BrY concentrations are shown in dashed lines. Columns not sharing a common superscript are significantly different (*P* < 0.05).

**Figure 4:**
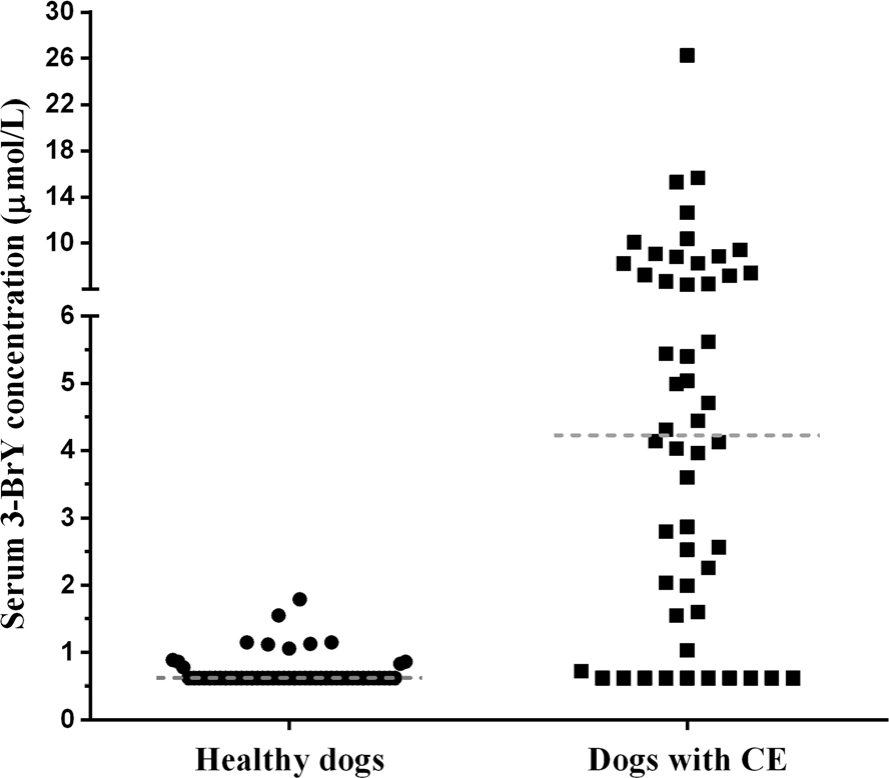
**Scatter plot of serum 3-BrY concentrations in 52 healthy dogs and 52 dogs with CE.** The medians for 3-BrY concentration are shown in dashed lines.

There was no significant association between peripheral eosinophil count and the presence of eosinophils in the GI tissues (*P* = 0.1733; Figure 2). In healthy dogs, the median serum 3-BrY concentration was ≤0.63 μmol/L with a range of ≤0.63 to 1.79 μmol/L. The median [range] of serum 3-BrY concentrations in dogs with EGE, LPE, EPI, and pancreatitis were 5.04 [≤0.63-26.26], 3.60 [≤0.63-15.67], 0.73 [≤0.63-4.59], and 1.49 [≤0.63–4.46] μmol/L, respectively. There was a statistically significant difference in serum 3-BrY concentrations between dogs with EPI, pancreatitis, LPE, EGE, and healthy dogs (*P* < 0.0001; Figure 3). The Dunn’s post-test analysis revealed differences of serum 3-BrY concentrations between healthy control dogs and dogs with either EGE, LPE, or pancreatitis. Serum concentrations of 3-BrY were significantly higher in dogs with EGE than in healthy dogs (*P* < 0.0001) or dogs with EPI (*P* = 0.0072). Also, serum 3-BrY concentrations were significantly higher in dogs with LPE than those in healthy dogs (*P* < 0.0001) or dogs with EPI (*P* = 0.0039). Serum 3-BrY concentrations were significantly higher in dogs with pancreatitis than in healthy dogs (*P* < 0.0135). However, there was no statistically significant difference of serum 3-BrY concentrations between dogs with EGE and LPE (P = 1.0000). Finally, Serum 3-BrY concentrations were significantly higher in dogs with CE (median [range]: 4.23 [≤0.63-26.26] μmol/L) than in healthy dogs (*P* < 0.0001; Figure 4).

## Discussion

The stability of 3-BrY concentrations in serum was evaluated in the present study because serum 3-BrY concentration may be affected by storage conditions. The results from this study suggest that 3-BrY serum concentrations are stable when stored at 4°C for ≤8 days, at −20°C for ≤30 days, and at −80°C for ≤180 days. Therefore, shipping and storage conditions require careful consideration.

Serum 3-BrY concentrations were slightly altered on day 8 when stored at 4°C and day 60 when stored at −80°C; however, this difference was not statistically significant, suggesting that these findings might also have been due to inter-assay variation. The %CV for inter-assay variation of serum 3-BrY concentration has previously been reported as ≤11.0% [15], whereas the %CVs for the storage conditions at 4°C and −80°C were ≤8.5 and 9.9%, respectively. Thus, we concluded that the alterations of serum 3-BrY concentrations in samples stored at 4°C and −80°C were most likely an effect of inter-assay variation.

The limitation of the stability study may be the fact that serum samples were pooled and a known concentration of 3-BrY had been added due to the fact that not enough serum with higher serum 3-BrY concentrations were available. Therefore, a known concentration of 3-BrY was added to each serum sample to reach the quantification limit where 3-BrY was detectable in all serum samples.

This study provides the first clinical evaluation of serum 3-BrY concentrations in dogs with GI diseases. No relationship between peripheral eosinophil count and the presence of eosinophils infiltration in the GI tissues was found in this study. This finding may be explained by the fact that eosinophils predominantly reside in the tissue instead of circulating in the blood stream. Therefore, peripheral eosinophils count are not always associated with eosinophil activation in the GI tissues [19,20]. Thus, the peripheral eosinophil count should not be considered as a minimally invasive marker for eosinophil activation in dogs with GI disease.

Lymphocytes and neutrophils have been reported as the predominant inflammatory cells present in dogs with pancreatitis [21]. In this study, serum 3-BrY concentrations were significantly increased in dogs with pancreatitis compared to healthy dogs. This was an unexpected finding, suggesting eosinophil activation in dogs with pancreatitis. This may suggest that pancreatic proteases are released and activate protease-activated receptor-2, which plays a role in the inflammatory pathway and activates eosinophils [22-24].

Our findings revealed a significant increase of serum 3-BrY in dogs with LPE and EGE, suggesting an important pathophysiological role of eosinophil activation in dogs with these two forms of CE. Serum 3-BrY concentrations for both dogs with EGE or LPE were statistically significantly different from healthy dogs. Although the median 3-BrY concentration in dogs with EGE was higher than in dogs with LPE, there was no statistically significant difference between these two groups. These findings suggest the presence of eosinophil activation in the GI tract of dogs with LPE or EGE. However, a larger sample set may have been needed to determine a possible difference in serum 3-BrY concentrations between dogs with EGE and those with LPE.

The presence of eosinophilic infiltration in the GI tract in dogs with EGE supports the hypothesis that 3-BrY may serve as a potential biomarker for eosinophil activation. Our findings are the first to demonstrate that 3-BrY, a stable product of eosinophilic peroxidase, can be detected in serum samples from dogs with chronic GI disease. Other studies in humans and mice have revealed the important pathophysiological role of EPO in affected GI tissues [25,26]. The lack of CCECAI and CIBDAI scores due to the use of left-over serum samples precluded us to identify the relationship between the severity of clinical signs and serum 3-BrY concentrations. Therefore, further investigations of the relationship between serum 3-BrY concentration and the severity of clinical signs are needed and underway.

One limitation of this study was that the tissue samples were evaluated by different pathologists from multiple diagnostic centers in the USA. Therefore, variations in histopathology scoring between pathologists may exist. Consequentially, the relationship between the degree of inflammatory cell infiltration in the GI tract and serum 3-BrY concentrations could not be performed. It should also be noted that mild to moderate eosinophilic infiltration may be present on biopsies in dogs with LPE. More studies are needed to identify the association between the grading of eosinophilic infiltration of the GI tract of dogs with CE and serum 3-BrY concentrations.

The increased serum 3-BrY concentration in dogs with LPE was an unexpected finding in the present study. Lymphocytes and plasma cells play a central role in dogs with LPE. The increased concentration of serum 3-BrY in LPE may be the result of eosinophil activation stimulated by T-lymphocytes in dogs with chronic GI disease. It is known that T-lymphocytes secrete IL-5, which is a key mediator to moderate the maturation, migration, and activation of eosinophils [27,28]. Moreover, eosinophil infiltration may be overlooked during the assessment in the different compartments (i.e., duodenum, jejunum, ileum, or colon) of the GI tract and this may lead to the failure to detect the eosinophil infiltration in those tissues. In addition, the use of hematoxylin and eosin (H&E) stain may have led to failure to detect eosinophil activation [29,30] when compared to other methods such as immunohistochemical staining (e.g., EPO antibody). However, measurements of 3-BrY concentration were performed in serum samples for this study, which may not accurately characterize inflammation in the GI tract. Therefore, the development and analytical validation of this assay for the measurement of 3-BrY concentration in fecal samples is warranted and underway.

## Conclusions

This is the first study that measured serum 3-BrY concentrations in dogs with various GI diseases. Our results suggest that eosinophil activation occurs in dogs with EGE, LPE, or pancreatitis. The present study also suggests that measurement of serum 3-BrY concentration may serve as a potential diagnostic marker for dogs with CE.

## Abbreviations

3-BrY: 3-bromotyrosine
CCECAI: Canine chronic enteropathy clinical activity index
CE: Chronic enteropathy
CIBDAI: Canine inflammatory bowel disease activity index
cPLI: Canine pancreatic lipase immunoreactivity
cTLI: canine trypsin like immunoreactivity
CV: Coefficient of variation
D_3_-BrY: d_3_-bromotyrosine
EGE: Eosinophilic gastroenteritis
EI-GC/MS: Electron ionization gas chromatography/mass spectrometry
EPI: Exocrine pancreatic insufficiency
EPO: Eosinophil peroxidase
GI LAB: Gastrointestinal Laboratory
GI: Gastrointestinal
HOBr: Hypobromous acid
HPLC: High performance liquid chromatography
IBD: Inflammatory bowel disease
LPE: Lymphocytic-plamacytic enteritis
MtBSTFA: N-methyl-N-(t-butyldimethylsilyl)-trifluoroacetamide

## References

[1] Allenspach K, Wieland B, Gröne A, Gaschen F (2007). Chronic enteropathies in dogs: evaluation of risk factors for negative outcome. J Vet Intern Med.

[2] Procoli F, Motskula PF, Keyte SV, Priestnall S, Allenspach K (2013). Comparison of histopathologic findings in duodenal and ileal endoscopic biopsies in dogs with chronic small intestinal enteropathies. J Vet Intern Med.

[3] Washabau RJ, Washabau RJ, Day MJ (2013). Large intestine. Canine and feline gastroenterology.

[4] German AJ, Washabau RJ, Day MJ (2013). Small intestine. Canine and feline gastroenterology.

[5] Jergens AE, Schreiner CA, Frank DE, Niyo Y, Ahrens FE, Eckersall PD (2003). A scoring index for disease activity in canine inflammatory bowel disease. J Vet Intern Med.

[6] Craven M, Simpson JW, Ridyard AE, Chandler ML (2004). Canine inflammatory bowel disease: retrospective analysis of diagnosis and outcome in 80 cases (1995–2002). J Small Animal Pract.

[7] Hall EJ, German AJ, Steiner JM (2008). Inflammatory bowel disease. Small animal gastroenterology.

[8] Al-Haddad S, Riddell RH (2005). The role of eosinophils in inflammatory bowel disease. Gut.

[9] Lampinen M, Ronnblom A, Amin K, Kristjansson G, Rorsman F, Sangfelt P (2005). Eosinophil granulocytes are activated during the remission phase of ulcerative colitis. Gut.

[10] Mita H, Higashi N, Taniguchi M, Higashi A, Kawagishi Y, Akiyama K (2004). Urinary 3-bromotyrosine and 3-chlorotyrosine concentrations in asthmatic patients: lack of increase in 3-bromotyrosine concentration in urine and plasma proteins in aspirin-induced asthma after intravenous aspirin challenge. Clin Exp Allergy.

[11] Weiss SJ, Test ST, Eckmann CM, Roos D, Regiani S (1986). Brominating oxidants generated by human eosinophils. Science.

[12] Wu W, Chen Y, d'Avignon A, Hazen SL (1999). 3-Bromotyrosine and 3,5-dibromotyrosine are major products of protein oxidation by eosinophil peroxidase: potential markers for eosinophil-dependent tissue injury in vivo. Biochemistry.

[13] Shen Z, Mitra SN, Wu W, Chen Y, Yang Y, Qin J (2001). Eosinophil peroxidase catalyzes bromination of free nucleosides and double-stranded DNA. Biochemistry.

[14] Wu W, Samoszuk MK, Comhair SA, Thomassen MJ, Farver CF, Dweik RA (2000). Eosinophils generate brominating oxidants in allergen-induced asthma. J Clin Invest.

[15] Sattasathuchana P, Grützner N, Rangachari VR, Berghoff N, Thengchaisri N, Guard BC (2014). Analytical validation of a gas chronmatography/mass spectrometry method for the quantification of 3-bromotyrosine in dog serum. J Vet Intern Med.

[16] Gaut JP, Byun J, Tran HD, Heinecke JW (2002). Artifact-free quantification of free 3-chlorotyrosine, 3-bromotyrosine, and 3-nitrotyrosine in human plasma by electron capture–negative chemical ionization gas chromatography mass spectrometry and liquid chromatography–electrospray ionization tandem mass spectrometry. Anal Biochem.

[17] Hazen SL, Crowley JR, Mueller DM, Heinecke JW (1997). Mass spectrometric quantification of 3-chlorotyrosine in human tissues with attomole sensitivity: a sensitive and specific marker for myeloperoxidase-catalyzed chlorination at sites of inflammation. Free Radic Biol Med.

[18] Frost MT, Halliwell B, Moore KP (2000). Analysis of free and protein-bound nitrotyrosine in human plasma by a gas chromatography/mass spectrometry method that avoids nitration artifacts. Biochem J.

[19] Young KM, Meadows RL, Weiss DJ, Wardrop KJ (2010). Eosinophils and their disorders. Schalm's veterinary hematology.

[20] Zuo L, Rothenberg ME (2007). Gastrointestinal eosinophilia. Immunol Allergy Clin North Am.

[21] Newman SJ, Steiner JM, Woosley K, Williams DA, Barton L (2006). Histologic assessment and grading of the exocrine pancreas in the dog. J Vet Diagn Invest.

[22] Matej R, Housa D, Olejar T (2006). Acute pancreatitis: proteinase-activated receptor-2 as Dr. Jekyll and Mr. Hyde. Physiol Res.

[23] Shpacovitch V, Feld M, Hollenberg MD, Luger TA, Steinhoff M (2008). Role of protease-activated receptors in inflammatory responses, innate and adaptive immunity. J Leukoc Biol.

[24] Schmidlin F, Amadesi S, Dabbagh K, Lewis DE, Knott P, Bunnett NW (2002). Protease-activated receptor 2 mediates eosinophil infiltration and hyperreactivity in allergic inflammation of the airway. J Immunol.

[25] Forbes E, Murase T, Yang M, Matthaei KI, Lee JJ, Lee NA (2004). Immunopathogenesis of experimental ulcerative colitis is mediated by eosinophil peroxidase. J Immunol.

[26] Carlson M, Raab Y, Peterson C, Hallgren R, Venge P (1999). Increased intraluminal release of eosinophil granule proteins EPO, ECP, EPX, and cytokines in ulcerative colitis and proctitis in segmental perfusion. Am J Gastroenterol.

[27] Takatsu K, Tominaga A, Hamaoka T (1980). Antigen-induced T cell-replacing factor (TRF). I. Functional characterization of a TRF-producing helper T cell subset and genetic studies on TRF production. J Immunol.

[28] Yan BM, Shaffer EA (2009). Primary eosinophilic disorders of the gastrointestinal tract. Gut.

[29] Gomes P, Torres SM, Plager DA, Jessen CR, Lee JJ (2013). Comparison of three staining methods to identify eosinophils in formalin-fixed canine skin. Vet Dermatol.

[30] Protheroe C, Woodruff SA, de Petris G, Mukkada V, Ochkur SI, Janarthanan S (2009). A novel histologic scoring system to evaluate mucosal biopsies from patients with eosinophilic esophagitis. Clin Gastroenterol Hepatol.

